# Seroprevalence and Associated Factors of Camel and Human Brucellosis in Dire District, Southern Ethiopia: A One‐Health Perspective

**DOI:** 10.1002/vms3.70835

**Published:** 2026-02-07

**Authors:** Yihenew Getahun Ambaw, Diba Guyo Kosi, Shimelis Mengistu, Simegnew Adugna Kallu

**Affiliations:** ^1^ College of Veterinary Medicine Haramaya University Dire Dawa Ethiopia; ^2^ Department of Veterinary Medicine College of agricultural sciences Woldia University Woldia Ethiopia; ^3^ College of Agriculture School of Veterinary Medicine Borana University Borana Ethiopia

**Keywords:** camel, camel brucellosis, Ethiopia, human brucellosis, I‐ELISA

## Abstract

**Introduction:**

The public health and economic impact of brucellosis remain a significant concern in Ethiopia. The high seroprevalence rates observed in both camels and humans indicate the potential for cross‐species transmission, highlighting the risk of brucellosis spreading. However, there is limited evidence concerning the relationship between brucellosis prevalence in humans and camels in the pastoralist regions of Ethiopia.

**Methods:**

A cross‐sectional study was conducted among 390 camels and 390 camel farmers in the Dire district from November 2023 to March 2024, using multistage sampling. This study aimed to determine the seroprevalence of brucellosis in camel farmers and their camels. During screening and confirming the presence of brucellosis, a modified rose Bengal plate test (MRBPT) and an indirect enzyme‐linked immunosorbent assay (I‐ELISA) were used, respectively, as a test series.

**Result:**

Among 390 camels, 7.17%, 95% CI (4.99–10.22) and 4.36%, 95% CI (2.72–6.91) were seropositive for *Brucella* using MRPT and I‐ELISA, respectively. Among 390 camel farmers, *Brucella* seroprevalence was 7.69% (95% CI: 5.42–10.81) by MRBPT and 3.08% (95% CI: 1.75–5.35) by I‐ELISA. In camels, adult (OR: 5.59, 95% CI: 1.67–44.48), female (OR: 2.83, 95% CI: 1.54–12.81) and large herds (OR: 5.10, 95% CI: 1.27–20.49) were statistically significant risk factors for the seroprevalence of camel brucellosis. In humans, the presence of a positive animal in the household (OR: 5.07, 95% CI: 1.10–23.34) and camel farmers who consume raw milk (OR: 2.75, 95% CI: 1.51–5.21) were also statistically significant risk factors for the seroprevalence of brucellosis.

**Conclusion:**

The presence of a *Brucella*‐positive camel in households and the consumption of raw milk highlight shared exposure at the human–animal interface. In marginal areas of Ethiopia, such as the Dire district, camel herders often face challenges accessing public services and information regarding zoonotic diseases. Consequently, promoting preventive strategies and raising awareness about the public health effects of camel brucellosis are encouraged to decrease the impact of this zoonotic disease in pastoral communities.

## Graphical abstract

The prevalence of camel brucellosis was 4.36%. Being adult, female and large herds were significant risk factors for camel brucellosis. The prevalence of human brucellosis was 3.08%. The presence of a positive animal in the household and camel farmers who consume raw milk were significant factors for human brucellosis.

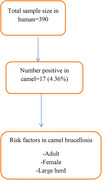

**Abbreviations**
ICC: interclass correlation coefficient
I‐ELISA: indirect enzyme‐linked immunosorbent assay
MOR: median odds ratio
MRBPT: modified rose Bengal plate test
YRVL: Yabello Regional Veterinary Laboratory

## 1 Introduction

Brucellosis is a neglected re‐emerging contagious bacterial disease that poses a significant threat to the One‐Health approach in pastoral regions (Fero et al. 2020). In the world, brucellosis ranks the second most significant zoonotic disease, after rabies (OIE 2009), which has around 2.1 million human cases reported each year (Laine et al. 2023). In camels, brucellosis causes significant financial losses by delaying initial calving, long calving interval, miscarriages, infertility, decreased milk output and mastitis (Kuplulu and Sarimehmetoglu 2004). The annual economic loss in livestock ranges from 200 to 600 million dollars (Edathodu et al. 2021).

Brucellosis can be transmitted to humans through the consumption of unprocessed animal products and contact with materials from animal births (Tschopp et al. 2021). The genus *Brucella* includes 12 species (Fero et al. 2020), such as *Brucella abortus* in cattle, *Brucella melitensis* in goats and camels, *Brucella suis* in pigs, *Brucella ovis* in sheep, *Brucella canis* in dogs and *Brucella neotomae* in rats, which are known as the classical variants (Godfroid et al. 2011). This disease can spread among animals and from animals to humans through direct contact or interaction with contaminated materials. Pastoralists are especially susceptible to contracting the disease due to their close physical interactions with animals and their cultural practice of consuming unpasteurized milk (Abbas and Agab 2002).

Brucellosis poses a significant challenge for diagnosis due to its nonspecific and often vague symptoms, which include a fever that follows a distinct pattern, night sweats, widespread bone pain, fatigue, joint pain, arthritis and lymph node swelling (Edathodu et al. 2021).

Factors contributing to the spread and persistence of brucellosis include animal movements, environmental conditions, the practices and behaviours of pastoralists, insufficient veterinary oversight, socio‐economic influences, the genetic make‐up of the host animals and the biology of *Brucella* species (Abutarbush 2010). Some factors that pose a risk for human brucellosis also include working with infected animals and the consumption of contaminated animal products (Donev et al. 2010).

Although brucellosis has been managed effectively in most developed countries, it continues to be the main socio‐economic, animal, and public health issue in developing nations, where large rural populations depend heavily on their livestock for income and food (Ducrotoy et al. 2014) and where there is a lack of resources and coordinated control initiatives (Tschopp et al. 2021).

The increased prevalence of brucellosis in pastoral areas is often linked to cultural and religious beliefs, as camel and livestock breeding play a significant role in pastoralists' traditions. In many pastoral areas, it is common for people to raise camels, sheep and goats, and commonly eat their meat and drink unpasteurized milk. These factors are all regarded as contributing risks for brucellosis (Al‐Homayani et al. 2023). Brucellosis is endemic in Ethiopia, with varying prevalence reported based on geographical location, farming practices and the species of animals involved (Tschopp et al. 2021). The seroprevalence of camel brucellosis in Ethiopia varies from 0.5% (Gessese et al. 2014) to 12.0% (Wakjira et al. 2022).

To design and implement a successful prevention and control program for zoonotic brucellosis in the pastoralist areas of Ethiopia, generating comprehensive evidence on the epidemiology of human and animal brucellosis is essential (Godfroid et al. 2011).

Despite its significant camel, public health and economic impacts for the previous decades, yet there is inadequate evidence on the integrated prevalence and associated factors of camels and camel farmers brucellosis in the pastoral communities, whose lives are highly connected to their animals and specifically in the Dire district, indicating the need for further study to develop effective control strategies and public health interventions. To address these gaps, an integrated seroprevalence of camels and camel farmers is needed. This study aimed to assess the integrated prevalence of human–camel brucellosis and its associated factors in the Dire district. The finding highlights the importance of a One‐Health framework to address the interconnected health of humans and animals concerning brucellosis in Ethiopia.

## 2 Material and Methods

### 2.1 Study Area

**FIGURE 1:**
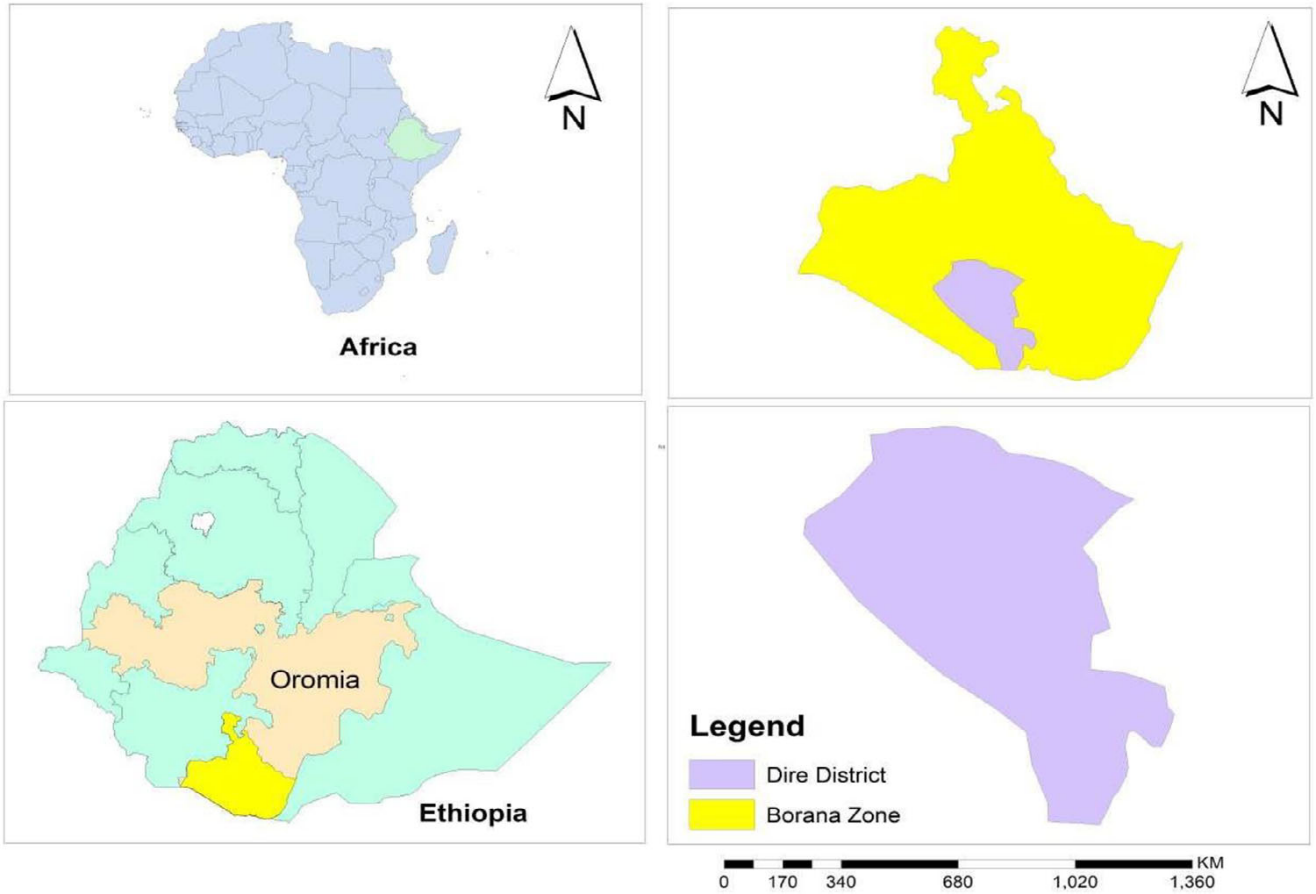
Maps of the study area.

The research was carried out in the Dire district located in the Oromia Region, specifically in the Borena Zone of Southern Ethiopia (Figure 1). Dire district has 11 villages and is found 675 km south of Addis Ababa and 100 km from Yabello, the capital city of the Borena Zone (DDPAO 2022). The total human population of the district is 55,642, including 28,043 males and 27,599 females. The district is found at an elevation of 750 m above sea level. The average monthly maximum and minimum temperatures in the district are 34°C and 19°C, respectively, and it receives a total annual rainfall of approximately 500 mm (CSADD 2022). The district is the residence of approximately 70,000 cattle, 150,000 goats, 101,000 sheep, 7,000 donkeys, 4,000 dogs and 40,000 camels. The veterinary infrastructure in the Dire district is quite inadequate, and each peasant association (PA) in the District has a single veterinary clinic. Over half of these health posts are not functional and out of service. This situation makes it challenging to provide veterinary services in the area, significantly hindering the delivery of animal healthcare in the Dire district (DDPAO 2022).

### 2.2 Study Design and Duration

A cross‐sectional study was done between November 2023 and March 2024. The study involved unvaccinated camels and their handlers from five PAs in the Dire district. Only camels older than 6 months were included in the study, as the disease was not prevalent in younger groups (Ambaw et al. 2025). Camels that had been vaccinated previously, those younger than 6 months, and owners unwilling to participate in the survey were excluded. The age of the sampled camels was determined by assessing their dental eruption and wear. Camels were categorized as follows: young (between 6 months and 7 years), adult (from 7 to 12 years) and old (over 12 years) (Bello et al. 2013). In addition, herd sizes are grouped into small (fewer than 10 camels), medium (11–25 camels) and large (more than 25 camels) (Ambaw et al. 2025).

### 2.3 Sample Size Determination and Sampling Procedures

In this study, a single population proportion formula, as outlined by Thrusfield (Thrusfield 2018), was used to estimate the minimum required sample size for camel–farmer pairs to assess the seroprevalence of camel and human brucellosis in the Dire district. Consequently, with an absolute precision of 5%, a confidence level of 95% and a previous seroprevalence of 12.0% (Wakjira et al. 2022), the calculation indicated a required sample size of 162 camel–farmer (human) pairs. However, the employed sampling method was multistage sampling, which accounted for a design effect of two and 20% contingency to address potential refusals from camels and camel farmers regarding participation in the survey. The total adjusted required sample size was 390 camels and 390 camel farmers.

The study examines the relationship between the seroprevalence of brucellosis in camels and humans at the household level. For this purpose, one camel–human pair was selected from each household. In the Dire district, there are eleven administrative PAs, and five of these were chosen using simple random sampling through random number generation in a Microsoft Excel spreadsheet. The primary sampling units were the PAs within the district. Villages within each PA served as the secondary units, and households (consisting of camel and camel–farmer pairs) within the villages were the tertiary units. Villages within the PAs were selected through cluster sampling in collaboration with veterinarians from those PAs. From each of the five selected PAs, two villages were chosen, resulting in the selection of 390 camel–human pairs (390 camels and 390 camel farmers) through cluster sampling among the ten selected villages.

### 2.4 Methods of Blood Sample Collection and Laboratory Analysis

Using a sterile plain vacutainer tube, blood samples of around 10 mL were obtained from each camel and human by restraining them, shaving the hair, and disinfecting the jugular vein sites. The samples were labelled and transported in an ice box to Yabello Regional Veterinary Laboratory (YRVL). After being kept at room temperature in a slanted position for 24 h, the serum was separated and centrifuged at 1500 rpm for 5 min at YRVL. Finally, the serum was carefully decanted into sterile cryovials, labelled, and stored in a refrigerator at −20°C until testing for brucellosis (Tekle et al. 2019). Modified rose Bengal plate test (MRBPT) was performed for screening purposes using a concentrated suspension of *B. melitensis*, Weybridge strain 99 (Institut Pourquier, France). The confirmatory test was conducted using the indirect enzyme‐linked immunosorbent assay (I‐ELISA) (ID Vet, 310, Innovative Diagnosis, France) at YRVL, following the testing protocols suggested by OIE (Afonso et al. 2012) and the specifications for the tests set by the production companies.

To perform the MRBPT on a glass plate, 75 µL of serum was combined with 25 µL of antigen suspension to carefully create a circular area measuring approximately 2 cm in diameter. This mixture was then shaken for about 4 min at room temperature (25°C). Samples that exhibited agglutination were regarded as positive for MRBPT, while those that did not were considered negative. In this instance, both negative and positive controls were used. The test's limitations included low sensitivity and specificity in chronic cases and endemic regions, respectively, alongside the possibility of biological false‐negative reactions. For the I‐ELISA test, 190 µL of dilution buffer was added to all wells, with 10 µL of negative control placed in wells A1 and B1, 10 µL of positive control in wells C1 and D1 and 10 µL of serum sample added to the remaining wells. Subsequently, the plate was incubated at 21°C for 45 min, followed by three washes of each well with 300 µL of wash solution. In each well, 100 µL of multispecies horseradish peroxidase conjugate was added, and the plate was incubated for 30 min at 21°C. After washing each well 3 times with 300 µL of wash solution again, the plate was kept in the dark for 15 min after adding 100 µL of substrate solution to each well. To halt the reaction, 100 µL of tetramethylbenzidine substrate (stop solution) was added in each well, and the optical density at 450 nm was subsequently measured and recorded for each sample. During recording, if the percentage of inhibition was ≥ 120, the result was categorized as positive; if it was between 110 and 120, it was deemed doubtful (uncertain), and if it was ≤ 110, it was classified as negative. Throughout this procedure, both negative and positive control sera were also included. Since the specificity and sensitivity of this test are not 100%, true seroprevalence can be calculated by considering the sensitivity and specificity of I‐ELISA (sensitivity, 0.93; specificity, 0.96). The limitation of I‐ELISA tests was the possibility of cross‐reactivity with secondary antibodies (Tschopp et al. 2021).

### 2.5 Data Management and Analysis

The collected raw data in the field were recorded in a Microsoft Excel spreadsheet (version 13). Descriptive statistics, including frequency and proportion, and inferential statistics, using the chi‐square test and multilevel binary logistic regression analyses, were executed using Stata software (version 17.0). Considering the hierarchical structure of the data inherent to the PA, which violates the independence assumptions of traditional binary logistic regression models, a multilevel binary logistic regression analysis was employed. Both bivariable and multivariable multilevel logistic regression analyses were conducted using an inter‐method model‐building approach, with variables having a *p* value of less than 0.25 included in the multivariable analysis. In the camel, all covariates examined in the bivariable analysis were incorporated into the final multivariable logistic regression model. In humans, variables such as age, family size and the status of the disposed aborted fetus were dropped from the analysis due to *p* values exceeding 0.25. The assessment of multicollinearity among the covariates was performed utilizing the variance inflation factor (VIF), with a VIF value exceeding 5 indicating potential multicollinearity. In this study, none of the covariates exhibited a VIF of more than 5. Three distinct models were employed: the null model (I), which contained no explanatory variables; the full model (II), a mixed model incorporating all candidate variables and the traditional multivariable logistic model, which encompassed all explanatory variables. For the mixed models, the interclass correlation coefficient (ICC) and the median odds ratio (MOR) were calculated to evaluate clustering effects in level two variables (PA). The deviance statistic (−2LL) and log‐likelihood values were used for model comparison. Among the three constructed models, Model II is the best‐fitted one, as evidenced by its lower deviance and log‐likelihood values. The association between covariates and the seroprevalence of brucellosis was determined by crude odds ratio. In the multivariable analysis, variables yielding *p* values below 0.05 were considered statistically significant factors associated with the prevalence of brucellosis in both humans and camels.

## 3 Result

In this report, the MRBPT test was used as the initial screening method due to concerns about the potential for false‐positive results. For final confirmation, the I‐ELISA test was employed because it is regarded as the most specific and sensitive serological test available. To assess the agreement between MRBPT and I‐ELISA, it is recommended that both tests be conducted in parallel on all samples. However, due to limited resources for purchasing I‐ELISA kits, only the samples that tested positive on the MRBPT were subsequently analysed using the I‐ELISA. In this study, the graphical and regression analyses relied on I‐ELISA test results.

### 3.1 Socio‐Demographic Information for Human Study Participants

Among 390 selected human participants, 51.3% were male, 39.0% were more than 50 years old, 20.0% were found in the Hododi PAs, 60.77% were married and 75.13% had not attended formal education (Table 1).

**TABLE 1:** Socio‐demographic characteristics of the human study participants at Dire district (*n* = 390).

Variables	Categories	Frequency	Percentage
Peasant association	Hododi	92	23.6
	Samaro	92	23.6
	Dololo	92	23.6
	Harallo	68	17.4
	Madacho	46	11.8
Sex	Male	103	26.4
	Female	287	73.6
Age	13–19	102	26.1
	20–49	136	34.9
	> 50	152	39.0
Educational status	No formal education	293	75.13
	Elementary	56	14.36
	High school	41	10.51
Marital status	Single	153	39.23
	Married	237	60.77
Family size	< 5	106	27.18
	≥ 5	284	72.82

### 3.2 Demographic Characteristics Among Camel Study Participants

Out of 390 selected camels for serological study, 73.6% were female, 40.5% were adult, 34.9% had a medium body condition score, and 41.3% had recruited from a medium herd size (Table 2).

**TABLE 2:** Univariable and multivariable multilevel binary logistic regression analysis among camel brucellosis (*n* = 390), at Dire district.

Variables	Categories	No examined (%)	No positive (%)	cOR	aOR	*p* value
Sex	Male	103 (26.4)	2 (1.9)	ref		
	Female	287 (73.6)	15 (5.2)	3.59 (0.77–16.58)	2.83 (1.54–12.81)	0.048^*^
Age	Young	102 (26.2)	1 (0.98)	ref		
	Adult	158 (40.5)	10 (6.3)	6.36 (0.79–51.11)	5.59 (1.67–44.48)	0.031^*^
	Old	130 (33.3)	6 (4.6)	4.66 (0.54–39.77)	2.60 (0.28–23.53)	0.394
Body condition	Poor	112 (28.7)	7 (4.9)	1.24 (0.41–3.75)	2.23 (0.56–8.84)	0.256
	Medium	136 (34.9)	3 (2.2)	0.44 (0.11–1.75)	0.58 (0.14–2.47)	0.460
	Good	142 (36.4)	7 (4.9)	ref		
Herd size	Small	148 (37.9)	4 (2.7)	ref		
	Medium	161 (41.3)	5 (3.1)	1.18 (0.31–4.53)	1.05 (0.24–4.54)	0.945
	Large	81 (20.8)	8 (9.9)	4.19 (1.13–15.46)	5.10 (1.27–20.49)	0.022^*^

Abbreviations: aOR, adjusted odds ratio; cOR, crude odds ratio; ref, reference.

^*^Statistically significant.

### 3.3 The Overall Seroprevalence of Camel Brucellosis With Its Associated Factors

**FIGURE 2:**
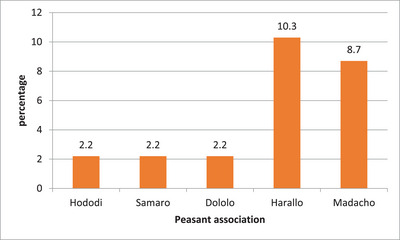
The prevalence of camel brucellosis across the peasant associations in Dire district.

In camels, the prevalence of brucellosis was higher (10.3%) in Harallo PA, followed by Madacho (8.7%) (Figure 2).

The total seroprevalence of camel brucellosis in Dire district was 7.17%, 95% CI (4.99–10.22) and 4.36%, 95% CI (2.72–6.91) by using MRPT and I‐ELISA, respectively. The multivariable binary logistic regression analysis demonstrated that female, large herd and adult were significantly associated with the seroprevalence of camel brucellosis (*p* < 0.05). The presence of *Brucella* antibody in female camel serum was 2.83 times (OR: 2.83, 95% CI, 1.54–12.81) more likely compared to males. The chance of getting *Brucella* antibody in large herd camel serum was also 5.10 times (OR: 5.10, 95% CI, 1.27–20.49) more likely relative to small herd camel. The odds of developing brucellosis in adult camels were 5.59 times (OR: 5.59, 95% CI, 1.67–44.48) more likely than in young camels (Table 2).

### 3.4 The Overall Seroprevalence of Human Brucellosis With Its Associated Factors

The total seroprevalence of human brucellosis in Dire district was 7.69%, 95% CI (5.42–10.81) and 3.08%, 95% CI (1.75–5.35) by using MRPT and I‐ELISA, respectively. The multivariable binary logistic regression analysis revealed that individuals who have positive animal at household and those who drink raw camel milk were significantly associated with the seroprevalence of human *Brucella* (*p* < 0.05). Thus, the seroprevalence of brucellosis in individuals who have positive animals in their household was 5.07 times (OR: 5.07, 95% CI, 1.10–23.34) more likely compared to their counterparts. The odds of developing *Brucella* antibody in individuals who drink raw camel milk were 2.75 times (OR: 2.75, 95% CI, 1.51–5.21) more likely relative to individuals who did not drink raw milk (Table 3).

**TABLE 3:** Univariable and multivariable multilevel binary logistic regression analysis in human brucellosis (*n* = 390), at Dire district.

Variables	Categories	No examined (%)	No positive (%)	cOR	aOR	*p* value
Sex	Male	200 (51.3)	8 (4.00)	ref		
	Female	190 (48.7)	4 (2.11)	0.44 (0.13–1.52)	0.34 (0.08–1.34)	0.123
Positive animal at household	Yes	24 (6.2)	4 (16.67)	8.58 (2.18–33.76)	5.07 (1.10–23.34)	0.037^*^
	No	366 (93.9)	8 (2.19)	ref		
Dispose aborted fetus	Yes	284 (72.8)	10 (3.52)	1.80 (0.37–8.74)		
	No	106 (27.2)	2 (1.88)	ref		
Age in years	13–19	102 (26.1)	4 (3.92)	ref		
	20–59	136 (34.9)	4 (2.94)	1.06 (0.24–4.63)		
	> 76	152 (39.0)	4 (2.63)	1.03 (0.23–4.70)		
Assisting during birth	Yes	322 (82.6)	8 (2.48)	ref	0.45 (0.10–1.98)	0.293
	No	68 (17.4)	4 (5.88)	2.86 (0.79–10.35)		
Consume raw milk	Yes	310 (79.5)	11 (3.55)	2.80 (1.52–4.25)	2.75 (1.51–5.21)	0.042^*^
	No	80 (20.5)	1 (1.25)	ref		
Family size	< 5	106 (27.18)	4 (3.77)	ref		
	≥ 5	284 (72.82)	8 (2.82)	0.68 (0.19–2.38)		

Abbreviations: aOR, adjusted odds ratio; cOR, crude odds ratio; ref, reference.

^*^Statistically significant.

**FIGURE 3:**
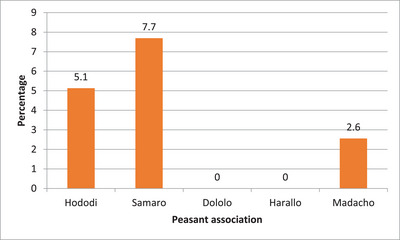
The prevalence of human brucellosis across the peasant associations in Dire district.

The majority, 289 (74.1%), of camel farmers have information about the zoonotic importance of camel brucellosis. Higher prevalence of human brucellosis was found in Samaro PA (7.7%), followed by Hododi (5.1%) and Madacho (2.6%) (Figure 3).

### 3.5 Random‐Effect Model and Model Fitness in Camel and Human Data

The random‐effect model in this cluster data analysis was evaluated using the ICC and MOR metrics. In the null modell, ICC values of 0.24 for humans and 0.09 for camels indicated that 24% and 9% of the total variation in *Brucella* seroprevalence among humans and camels, respectively, occured between clusters or PAs. Moreover, the higher MOR values of 2.66 for humans and 1.72 for camels indicate a significant degree of clustering of brucellosis cases within these groups. In both humans and camels, the full model, which incorporates candidate variables, proved to be the best‐fitting model, as evidenced by its lowest deviance statistics (Table 4).

**TABLE 4:** Comparison of model fit statistics for Brucella seroprevalence in camels and humans.

Parameter	Null model (mixed)		Full model (mixed)		Traditional logistic model	
	Camel	Human	Camel	Human	Camel	Human
ICC	0.09	0.24	0.07	0.12		
MOR	1.72	2.66	1.74	1.88		
Model comparison						
Log‐likelihood	−69.09	−52.14	−61.03	−42.58	−60.84	−41.99
Deviance	138.18	104.28	122.06	85.16	121.68	83.98

An MOR of 1.72 in camels indicates significant clustering of brucellosis (median heterogeneity) when randomly selecting two camels from different clusters (PAs).

## 4 Discussion

This research was conducted by employing a One‐Health approach to concurrently examine the occurrence of brucellosis in both humans and livestock residing in the same households. While animals serve as the primary reservoirs for human brucellosis, investigations into this disease have largely been carried out independently for humans and animals (Ducrotoy et al. 2014).

Among the whole 390 camel owners, approximately three‐fourths (74.10%) knew that brucellosis can affect humans. This finding was nearly comparable to the reports in Saudi Arabia (Al‐Homayani et al. 2023), which showed that 79.6% of the study participants had information that humans were impacted by brucellosis. On the other hand, studies conducted in Ethiopia, 4% (Gessese et al. 2014), in Kenya, 89% (Njenga et al. 2020) and in Tibet, 53.7% (Zeng et al. 2018), revealed that respondents knew that brucellosis affects humans. The variation in the level of knowledge about brucellosis might be associated with the difference in the level of education and availability of information among the respondents in different study areas.

The total seroprevalence of camel brucellosis in the Dire district was 4.36%, 95% CI (2.72–6.91), by using the I‐ELISA test. The employed diagnostic tests in this study have only been validated for humans (Afonso et al. 2012) and other livestock species, including cattle, goats and sheep; consequently, interpreting the results for camels requires cautious consideration (Sibhat et al. 2024). The 4.36% seroprevalence of brucellosis in camels is equivalent to the study conducted in the Fafan zone, 4.9% (Lakew et al. 2019), in Amibara and Dubti districts, 5.4% (Sibhat et al. 2024) and in Saudi Arabia, 5.28% (Almuzaini et al. 2025). These comparable prevalence reports can be attributed to the similar agro‐ecological and animal management systems in the study areas. The agro‐ecological conditions of these study regions are characterized as dry and semidry, facing challenges with feed and water shortages, and animals are managed using extensive systems that involve shared grazing and watering points. The diagnostic tests used in these studies included MRBT and I‐ELISA, which allow for nearly comparable results (Hussen et al. 2023).

However, the current finding was higher than the reports in Adama city, 0.53% (Gessese et al. 2014), in Adadle district, 0.6% (Ibrahim et al. 2021), in and around Dire Dawa, 2% (Waktole et al. 2022) and in Oman, 0.4% (Alrawahi et al. 2019). On the other hand, the present report was lower than the findings in Borana, 12.44% (Wakjira et al. 2022) and in Qatar, 20.6% (Alhussain et al. 2022). This disparity in the seroprevalence of camel brucellosis observed in the present study compared to previous reports may be attributed to differences in animal management practices and environmental conditions. In addition, the annual rainfall in the most affected districts is very minimal, leading to a shortage of water and feed in the region; consequently, camels are compelled to migrate in search of food and water, which raises the chances of direct or indirect interactions among animals and may enhance the risk of brucellosis spread. Moreover, currently, there is no vaccination program for brucellosis in the study area, which worsens the condition (Ahmed 2021). In the Dire district, the combination of pastoralism and communal grazing practices, along with limited access to veterinary and public health services, leads to camel brucellosis becoming endemic (Racloz et al. 2013).

The study revealed that age was a risk factor for the prevalence of camel brucellosis. Adult camels were more likely to be infected compared to young animals. This finding was supported by earlier reports by Wubaye et al. (2024), Hussen et al. (2023) and Edao et al. (2020). This may occur since animals might contract *Brucella* when they are young, the bacteria settle in the regional lymph nodes without triggering antibody production until the animals attain sexual maturity and start producing sex hormones and erythritol, which promote the growth and proliferation of *Brucella* species (Alrawahi et al. 2019).

Herd size was also a significant risk factor for the prevalence of camel brucellosis. Camels found in larger herds in the Dire district show a higher rate of seropositivity than those in smaller herds. This observation does not align with findings from other studies (Sibhat et al. 2024; Alrawahi et al. 2019). The variation in seroprevalence could be attributed to higher stocking densities in large herds, which may facilitate the spread of brucellosis, particularly during calving or abortion periods. In addition, this difference may be influenced by fluctuations in disease prevalence at the overall animal level and within the herd size during the study timeframe (Ahad et al. 2024). The increased incidence of camel brucellosis in pastoralist regions, in contrast to mixed crop–animal production areas in Ethiopia, could be linked to animal movements and the management of large herds in these pastoral systems, which create opportunities for close contact among potential sources of infection and numerous susceptible animals in tightly confined spaces (Asmare et al. 2013).

In terms of sex, female camels showed a higher likelihood of being exposed to brucellosis compared to males. This observation aligns with findings from other studies conducted in various regions (Edema et al. 2025). The increased occurrence of brucellosis in female camels may be linked to the hormone erythritol, a sugar alcohol found in higher concentrations in the placenta and fetal fluids of pregnant animals, thereby creating an environment conducive to the growth and reproduction of *Brucella* organisms. Females have higher levels of erythritol sugar than males, which raises their susceptibility to brucellosis (Radostits et al. 2007). On the other hand, the elevated seroprevalence of *Brucella* in female camels could also be attributed to age; since most male camels are sold at a young age for meat, the population consists of more young males than adult ones. Even if young animals become infected with *Brucella*, the bacteria tend to reside in the regional lymph nodes without triggering antibody production until the animals reach sexual maturity and begin producing sex hormones that promote the growth and multiplication of *Brucella* (Radostits et al. 2007). In this research, the proportion of female camels (73.6%) was greater than that of males. Typically, pastoral herds consist of more females than males, as female animals are retained longer for their milk production, which increases their likelihood of being exposed to brucellosis. In contrast, males are primarily kept for financial gain or meat. This situation may also amplify the risk of disease transmission from animals to humans, as pastoralists consume raw milk and meat from these female camels (Tschopp et al. 2021).

The overall seroprevalence of human brucellosis in the Dire district was found to be 3.08%, with a 95% CI of (1.75–5.35), as determined by the I‐ELISA test. This finding was in line with other reports in the Amhara region, 3.0% (Genene Regassa et al. 2009), in the Oromia region, 3.6% (Tolosa et al. 2007) and in the Oromia region, 2.6% (Edao et al. 2020). On the other hand, this finding was lower than other reports in Afar, 48.3%, in the Somali region, 34.9% (Tschopp et al. 2021), in Oromia, 34.1% (Genene Regassa et al. 2009), in the Southern region, 29.2% (Genene Regassa et al. 2009), and a systematic review and meta‐analysis in Ethiopia, 14% (Asrie et al. 2025). The discrepancies in prevalence across various studies may be linked to differences in geographic regions of the study undertaken, the diagnostic techniques used to detect the agent, the health policies employed in different locations, the livestock practices of the communities, the difference in sample size included in each reports, the lifestyle of the society, and the socio‐economic circumstances of the participants involved in the studies (Asrie et al. 2025).

Besides, the potential reasons for the higher prevalence of the disease in Afar and Somali regions could be the limitation of knowledge regarding brucellosis, poor dietary choices such as intake of unpasteurized dairy or undercooked meats, conventional livestock rearing methods and inadequate monitoring and vaccination initiatives. For instance, in the Afar region of Ethiopia, over 80% of the inhabitants rely on pastoral livelihoods, devoting significant time to their livestock. Their main sources of food are animal‐derived products, especially milk, which is frequently consumed without boiling. The intake of unpasteurized or insufficiently cooked animal products is a significant factor in the spread of brucellosis from animals to humans (Asrie et al. 2025).

When we see the association between the presence of a positive animal and the seroprevalence of brucellosis in the same household, camel owners with a positive animal in their house were 5 times more likely compared to owners who had no positive animal in their household. The finding is in agreement with the report of Osoro et al. (2015) and Bonfoh et al. (2012), which suggests that the likelihood of human brucellosis was higher in households with seropositive animals. The highest odds were observed in households with camels that tested positive for *Brucella*. Previous reports showed a significant correlation between seropositivity in humans and animals living in the same house. In particular, a strong correlation was observed between human seropositivity and that of goats and camels (Osoro et al. 2015). In contrast, it highlights findings from research conducted in Togo and Mongolia, where no correlation was identified between human and animal seropositivity (Zolzaya et al. 2014; Dean et al. 2013). However, it is important to note that sampling in Togo was conducted at the PA level instead of the household level. The research in Mongolia showed no link between cattle and human seropositivity and identified a significant correlation only with sheep, but not in goats. In that study, the sampling occurred at the PA level, and livestock and human samples were not always taken from the same households (Zolzaya et al. 2014). Authors also believe that the variation in the sampling level may explain the discrepancies noted in their report (Osoro et al. 2015).

Regarding raw milk consumption, individuals who drink raw milk were 2.73 times more likely to develop brucellosis compared to those who do not. This finding is in agreement with an earlier study conducted in Kenya (Osoro et al. 2015), which indicated that people who regularly consume raw milk are 3.5 times more likely to develop brucellosis than those who do not drink. The high prevalence of brucellosis among those who consume raw milk can be attributed to the intake of raw milk and unpasteurized dairy products that are infected with *Brucella* bacteria. The disease‐causing agent is present in the milk secretions of infected animals; the ingestion of milk and unpasteurized dairy products in regions where *Brucella* prevalent is a primary method of transmitting brucellosis to people. Numerous studies have confirmed the existence of *Brucella* species in milk and unpasteurized dairy items, and adequate heating can eradicate these pathogens; therefore, pasteurizing milk and other dairy products can significantly reduce the risk of this disease in humans (Abdali et al. 2020; Izadi et al. 2014).

## 5 Limitations of the Study

To evaluate the correlation between MRBPT and I‐ELISA, it is advisable to perform parallel tests for all samples; however, due to budget constraints limiting the purchase of I‐ELISA kits for the entire sample set, only those samples that tested positive in MRBPT underwent I‐ELISA evaluation. The serological tests used in this study are primarily approved for humans and common farm animals (cattle, goats, and sheep); therefore, interpreting the results for camels should be in caution.

## 6 Conclusion

The present study revealed a significant prevalence of brucellosis among humans and camels in the Dire district. Being an adult, having a large herd, and being female were significantly associated with camel brucellosis, whereas, in humans, the key risk factors were having a positive animal in the household and being camel farmers who consume raw milk. Therefore, brucellosis poses a serious public health threat to both camels and humans in the Dire district. It is crucial to promote behavioural changes concerning animal management, mainly regarding their discharges during abortion and parturition. Moreover, camel farmers are encouraged to consume boiled milk and well‐cooked meat to reduce the infection rate. Comprehensive epidemiological surveys that adopt a One‐Health approach are required to detect the circulating species of *Brucella* in animals and humans in the Dire district.

## Author Contributions

**Yihenew Getahun Ambaw**: conceptualization, investigation, methodology, data curation, formal analysis, writing – original draft. **Diba Guyo Kosi**: conceptualization, investigation, methodology, writing – original draft. **Shimelis Mengistu**: data curation, supervision, writing – review and editing. **Simegnew Adugna Kallu**: methodology, formal analysis, writing – review and editing.

## Funding

The authors have nothing to report.

## Ethics Statement

Ethical approval for this study was obtained from Haramaya University, College of Veterinary Medicine Ethical Clearance Review Committee (Ref. No.: CVM/578/2023). Informed consent was obtained from all study participants, who demonstrated their agreement to participate by signing the consent forms. In this study, individuals aged 18–65 years were eligible for inclusion; therefore, assent forms were not necessary.

## Conflicts of Interest

The authors declare no conflicts of interest.

## Data Availability

The data that support the findings of this study are available from the corresponding author upon request.
